# A Single-Center Analysis of Patient Characteristics and Overall Survival in Patients with Resectable Gallbladder Cancer

**DOI:** 10.3390/healthcare12202091

**Published:** 2024-10-21

**Authors:** N. Begüm Öztürk, Artem Dadamyan, Laith H. Jamil

**Affiliations:** 1 Department of Internal Medicine, Corewell Health William Beaumont University Hospital, Royal Oak, MI 48073, USA; 2 Oakland University William Beaumont School of Medicine, Rochester, MI 48309, USA; adadamyan@oakland.edu (A.D.); laith.jamil@corewellhealth.org (L.H.J.); 3 Section of Gastroenterology and Hepatology, Corewell Health William Beaumont University Hospital, Royal Oak, MI 48073, USA

**Keywords:** gallbladder neoplasm, biliary cancer, cholecystectomy

## Abstract

Introduction: Gallbladder cancer (GBC) is a rare and aggressive hepatobiliary malignancy with poor prognosis. The symptoms of GBC are insidious and non-specific in its early stages, and most patients are diagnosed at advanced or late stages. Surgical resection is the only potentially curative treatment for GBC for select patients. There is a lack of robust data for patients with GBC, leading to heterogenous practices in management strategies and outcomes. In this study, we aimed to identify patient characteristics and cumulative overall survival (OS) in patients with GBC who underwent surgical resection with curative intent. Methods: All adult patients (age ≥18 years) with localized or locoregionally advanced GBC who underwent definitive surgery with curative intent at our tertiary institution between 1/2013 and 12/2023 were retrospectively identified. Clinical, laboratory, radiology, histopathology, treatment, and survival data were collected from electronic medical records. Postoperative data included the use of adjuvant chemotherapy or radiotherapy, and patient survival mortality at a cut-off date of 1 February, 2024, calculated from the date of curative surgery. Continuous variables are reported as median and quartile 1 (Q1) and quartile 3 (Q3), while categorical variables are reported as counts and percentages. Results: A total of 94 patients with GBC were included in the study. Median age was 71 (62–77) years and 58 (61.7%) patients were female. Median tumor size was 3.3 (1.9–5.0) cm. Perineural invasion was seen in 48.9% and vascular invasion in 38.3% of patients. A positive surgical margin was present in 50% of the patients, and incidental GBC (IGBC) was seen in 48.9% of patients. Tumor grade was well differentiated in 7.6%, moderately differentiated in 53.3%, and poorly differentiated in 39.1% of the patients. Patients with stage T1a (2.1%) and T1b (11.7%) tumors comprised the minority, and the majority of the tumors were stage T2 (55.3%), followed by T3 (31.9%). A total of 60.6% of patients with GBC underwent adjuvant chemotherapy, and 17% underwent adjuvant radiotherapy after surgical resection. Overall, 62 (66.0%) patients died, and the median OS was 1.88 years. The 1-year OS was 68.7%, 3-year OS was 37.4%, and 5-year OS was 32.2%. A higher absolute median OS was seen in patients who had adjuvant chemotherapy (2.1 years) compared to no chemotherapy (1.9 years); however, this finding was not statistically significant (*p* = 0.36). The median survival was 2.3 years in IGBC compared to 1.6 years in non-IGBC (*p* = 0.63). Conclusions: GBC is an aggressive hepatobiliary malignancy that is often diagnosed at advanced stages. Our study showed high rates of local and systemic involvement and high mortality, and the need for prospective and randomized studies on adjuvant therapies to assess their survival benefit. Real-world patient data remain important to identify patients at risk of worse outcomes and to stratify risks prior to surgery.

## 1. Introduction

Gallbladder cancer (GBC) is a rare and aggressive tumor with poor prognosis [1,2,3]. The epidemiology of GBC exhibits wide geographic variations due to a combination of predisposing factors including genetics, family history, sex, chronic infection and inflammation, and congenital developmental anomalies [2,4]. The risk factors for GBC have been described as the presence of gallstones, a larger size or longer duration of gallstones, gallbladder calcification, gallbladder polyps (especially ≥10 mm), congenital biliary cysts, older age, female sex, obesity, chronic infections (*Salmonella*, *Helicobacter*), and carcinogen exposure [2,5]. 

The US has a lower GBC incidence, with a rate of 1.4 per 100,000 among females and 0.8 among males, compared to countries in South America, such as Bolivia (12.8 for males, 15.1 for females per 100,000), and South and East Asia, such as Nepal (6.0 for males, 7.3 for females per 100,000) and India (2.3 per 100,000), which experiences one of the highest GBC incidence rates globally [2,6,7,8,9]. In the US, approximately two thirds of GBC cases and GBC deaths were reported to occur among females [10]. The incidence of GBC has been stable over the past two decades in the majority of populations in the US, except non-Hispanic Blacks [2,11]. Approximately 20% of GBC cases are diagnosed at an early stage in the US, and the overall average 5-year survival has been reported as 18% [2,11]. 

The symptoms of GBC are insidious at the early stage of the disease, and approximately 50–70% of GBCs are diagnosed incidentally after laparoscopic cholecystectomy [12,13]. In the US, a study reported that 43% of GBC cases had spread to the regional organs or lymph nodes, and 42% had spread to the distant organs or lymph nodes, at the time of diagnosis [10]. Complete surgical resection is the only potentially curative treatment for localized or locoregionally advanced GBC. As GBC is often detected at advanced stages, the median survival for those undergoing resection is around 12–14 months, and less for patients with advanced disease [2]. 

Given the rarity of GBC, studies utilizing real-world patient data remain important to understand the patient characteristics, presentation, management, and outcomes. In this study, we aimed to identify patient characteristics and cumulative overall survival (OS) in patients with GBC that underwent surgical resection with curative intent at our institution. 

## 2. Methods

All adult patients (age ≥18 years) with localized or locoregionally advanced GBC who underwent definitive surgery with curative intent at our tertiary institution between 1/2013 and 12/2023 were identified and retrospectively analyzed. The STROBE checklist was followed for the study (Supplementary Materials). Institutional review board approval was obtained by our institution (#2023-034) and informed consent was waived due to the retrospective nature of the study. The study was performed in line with the principles of the Declaration of Helsinki. 

### 2.1. Inclusion and Exclusion Criteria

An initial search was conducted using the ICD-10 code C23 in the electronic medical records and a manual review was then performed to evaluate the true presence of GBC based on histopathological confirmation. Adult patients who underwent surgery with curative intent for incidental GBC (diagnosed following cholecystectomy for benign reasons) and non-incidental GBC (diagnosed pre-operatively) were included in the study. Histological classification was performed according to the guidelines of the most recent (8th edition) American Joint Committee on Cancer (AJCC) [14]. Patients who had GBC but did not undergo curative surgery and patients with cholangiocarcinoma (CCA), hepatocellular carcinoma (HCC), or combined HCC-CCA on histopathological evaluation were excluded. 

### 2.2. Demographic, Clinical, Pathological, and Survival Data Collection

Clinical, laboratory and imaging, histopathology, treatment, and survival data were collected from electronic medical records. All patients had abdominal ultrasound, contrast-enhanced computed tomography, or magnetic resonance imaging to complete staging prior to surgery. Tumor size, the grade of differentiation, surgical margin, the presence of vascular invasion, and perineural invasion were assessed on histology. Postoperative data included the use of adjuvant chemotherapy or radiotherapy. Patient survival was assessed with a cut-off date of 1 February, 2024, calculated from the date of curative surgery. 

### 2.3. Statistical Analysis

Continuous variables are reported as median and quartile 1 (Q1) and quartile 3 (Q3), while categorical variables are reported as counts and percentages. Continuous variables were compared using Student’s *t*-test or Mann–Whitney U test and categorical variables were compared using Chi-square or Fisher’s exact test, as appropriate. Overall survival was calculated from the date of cholecystectomy to the date of death or last clinical encounter. Time to death or censoring was analyzed using the Kaplan–Meier method with log rank-sum tests. *p* < 0.05 was considered significant for all analyses. Statistical analyses were performed using Prism 10 (San Diego, CA, USA). 

## 3. Results

**Figure 1:**
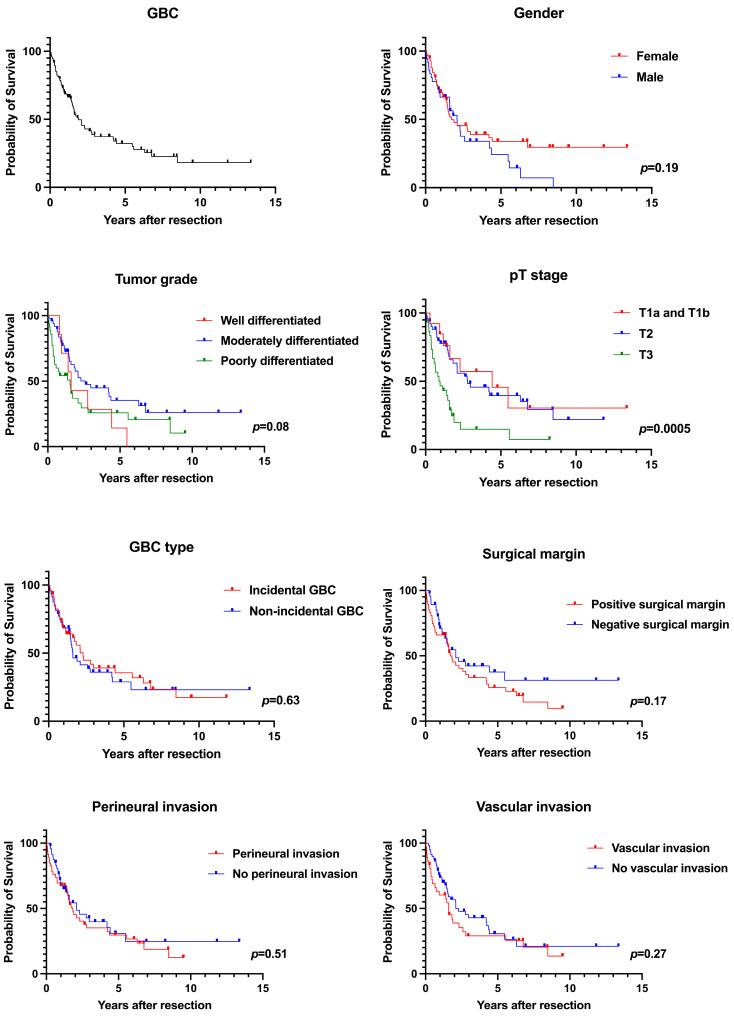
Kaplan-Meier survival analyses.

The initial search strategy identified 179 unique patients and a total of 94 patients were included in the study. Median follow-up duration was 1.5 (0.7–3.9) years. The median follow-up duration for patients who were alive but censored at the last visit was 3.4 (1.3–6.6) years, and the median follow-up duration for patients who died was 1.2 (0.5–2.1) years. Table 1 shows the patient characteristics. Median age was 71 (62–77) years, and 58 (61.7%) patients were female. The majority of the patients were White (75.5%) and the remaining 24.5% were Black. Median tumor size was 3.3 (1.9–5.0) cm. Perineural invasion was seen in 48.9% and vascular invasion in 38.3% of patients. Positive surgical margin was present in 50% of patients with GBC that underwent curative resection. Incidental GBC (IGBC) was seen in 48.9% of patients. The majority of tumor histologies were adenocarcinoma (96.8%). Tumor grade was well differentiated in 7.6%, moderately differentiated in 53.3%, and poorly differentiated in 39.1% of the patients. Patients with stage T1a (2.1%) and T1b (11.7%) comprised the minority, and the majority of the tumors were stage T2 (55.3%), followed by T3 (31.9%). Overall, the median OS was 4.4 years for T1a and T1b tumors combined, 2.8 years for T2 tumors, and 0.9 years for T3 tumors after curative surgery (*p* = 0.0005). During the study period, 62 (66.0%) patients died, with a median OS of 1.88 years. The 1-year OS was 68.7%, 3-year OS was 37.4%, and 5-year OS was 32.2%. Table 2 shows the OS of all study patients and Figure 1 shows the associated Kaplan–Meier curves. 

### 3.1. Sex Disparities in GBC

The median age at diagnosis of GBC was 69 (61–76) years in females compared to 72 (64–78) years in males (*p* = 0.48). There were no significant differences in tumor size, perineural and vascular invasion, the presence of cholelithiasis, gallbladder polyp, death rates, and the duration from surgical resection to death between both sexes. The utilization of adjuvant chemotherapy and radiotherapy was comparable in both groups. The median OS was not statistically significant, at 1.7 years in females and 2.1 years in males (*p* = 0.19). Overall, the 1-year survival rate was 70% in females compared to 66.3% in males, the 3-year survival rate was 39.1% in females compared to 34.0% in males, and the 5-year survival rate was 33.8% in females compared to 24.3% in males. 

### 3.2. Adjuvant Chemotherapy and Radiotherapy

A total of 60.6% of patients with GBC received adjuvant chemotherapy, and 17% received adjuvant radiotherapy after surgical resection. A total of 38.3% of the patients did not receive any neoadjuvant or adjuvant chemotherapy, or radiotherapy, and only had surgical resection. Only patients who received adjuvant chemotherapy also received adjuvant radiotherapy (28.1%). Patients who received adjuvant chemotherapy were significantly younger [69 (59–74)] compared to patients who did not receive adjuvant chemotherapy [75 (66–83)], *p* = 0.001. In addition, patients who received adjuvant chemotherapy more frequently had vascular invasion (45.6% vs. 27.0%, *p* = 0.016). There were no statistically significant differences in terms of sex, tumor size, or the presence of IGBC vs. non-IGBC (NIGBC) in patients who received adjuvant chemotherapy compared to patients who received no adjuvant or neoadjuvant chemotherapy. 

The 1-year OS rate was 80.1% in patients who received adjuvant chemotherapy compared to 51.1% in patients who did not, the 3-year OS rate was 38.6% in patients who received adjuvant chemotherapy compared to 34.8% in patients who did not, and 5-year OS was 28.3% in patients who received adjuvant chemotherapy compared to 29.1% in patients who did not. The duration from surgery to death was significantly longer in patients who received adjuvant chemotherapy [586 (317–974) days] compared to those who did not [208 (81–422) days], *p* = 0.019. 

### 3.3. Incidental vs. Non-Incidental GBC

IGBC was present in 48.9% of patients and NIGBC was present in 51.1% of patients with resectable GBC. Table 3 shows the characteristics of patients with IGBC and NIGBC. Cholelithiasis was significantly more common in patients with IGBC (91.3%) compared to NIGBC (66.7%), *p* = 0.002. The median survival was 2.3 years in patients with IGBC compared to 1.6 years in patients with NIGBC (*p* = 0.63). The 1-year OS rate was 68.9% in patients with IGBC compared to 68.5% in patients with NIGBC, the 3-year OS rate was 39.1% in patients with IGBC compared to 36.0% in patients with NIGBC, and the 5-year OS rate was 35.5% in patients with IGBC compared to 28.8% in patients with NIGBC. 

## 4. Discussion

GBC is an aggressive malignancy with poor prognosis and a median survival of 3–22 months [15]. GBC is often diagnosed at advanced stages, and complete surgical resection remains the only potential curative treatment in patients who are candidates for surgery [16,17]. In our study, we assessed the characteristics and the survival outcomes of patients with resectable GBC and reported our single, tertiary care experience. 

As reported by multiple other studies, GBC disproportionally affects females, and similarly, 61.7% of our cohort were females. In addition, gallbladder polyp, cholelithiasis, and overweight BMI were commonly seen in patients with GBC. There were no significant sex differences for vascular or perineural invasion at diagnosis, the stage or grade of the tumor, or the use of adjuvant chemotherapy or radiotherapy. Although there were no statistically significant differences in cumulative OS, females had a lower absolute median OS of 1.7 years after surgical resection, compared to 2.1 years in males. 

Adjuvant chemotherapy with gemcitabine and cisplatin is often used after surgical resection in GBC; however, there are no phase III randomized clinical trials evaluating this regimen in GBC. Regarding the use of capecitabine, the phase III BILCAP trial that included 18% of patients with GBC showed that patients treated with capecitabine had a higher absolute OS of 51.1 (95% CI, 34.6–59.1) months compared to 36.4 (95% CI, 29.7–44.5) months in the observation arm, without statistical significance [18]. In addition, there is a lack of high-quality evidence for the utility of neoadjuvant therapy for GBC and the use of neoadjuvant therapy for GBC is uncommon the US [19,20,21]. A large international multicenter retrospective study that included 3676 patients with GBC reported that the majority of centers utilized aggressive surgical approaches, and had low rates of the utilization of neoadjuvant (2.4%) or adjuvant (33%) chemotherapies [21]. The same study also reported that cholecystectomy alone was sufficient for most early-stage GBCs (T1a); however, extended resections were required for higher grade tumors, and for tumors with nodal involvement, extended resections were associated with increased mortality without definitely improving OS [21]. Another study utilizing the National Cancer Database reported that the use of adjuvant chemotherapy in resectable GBC was 49.2%, and the use of neoadjuvant chemotherapy was 1.6% [19]. Adjuvant or neoadjuvant chemotherapy were more commonly used in patients with lymph node involvement, and patients who had adjuvant chemotherapy had a significantly improved OS compared to those who received surgery alone (22 vs. 18 months, HR: 0.78, 95% CI: 0.63–0.96) according to the propensity score-matching analysis [19]. In our study, although there was no significant OS difference, the absolute median OS was higher in patients who had adjuvant chemotherapy (2.1 years) compared to those who received no adjuvant chemotherapy (1.9 years), *p* = 0.36. Moreover, our study found that the patients who received adjuvant chemotherapy were significantly younger and more frequently had vascular invasion. This observation could be explained by the presence of older patients in our cohort and adjuvant chemotherapy being offered only to patients with tumor invasion to the lymph nodes or vasculature. The tumor size, grade of differentiation, stage, and IGBC or NIGBC status were not associated with receiving adjuvant chemotherapy. Compared to the other studies, our cohort had a relatively higher absolute percentage of patients receiving adjuvant chemotherapy, which may be explained by our center being a tertiary center. 

Similarly to adjuvant chemotherapy, there are no randomized controlled trials (RCTs) regarding the use of adjuvant radiotherapy in patients with GBC. The recommendations for adjuvant radiotherapy are based on a phase II, small, single-arm SWOG S809 trial that included patients with extrahepatic cholangiocarcinoma and GBC, which reported an overall median survival of 35 months and a 2-year survival of 65% in patients who were treated with adjuvant radiotherapy after adjuvant capecitabine, a significant improvement compared to the historical controls [22]. Another retrospective cohort study including patients with T2/T3 GBC also demonstrated favorable survival outcomes in select patients (lymph node-positive T2/T3 GBC) who received adjuvant chemoradiotherapy [23]. Another study, conducted using the National Cancer Database, focusing on 4977 patients meeting the SWOG S0809 criteria, to evaluate the use of adjuvant chemoradiotherapy in a resected extrahepatic bile duct and GBC, reported a similar median OS, of 36.9 months, as SWOG S0809, and a 2-year survival rate of 65.6% in patients who received adjuvant radiotherapy along with chemotherapy [24]. In our study, adjuvant radiotherapy was utilized in 17% of patients, and adjuvant radiotherapy was only utilized in patients who were on adjuvant chemotherapy.

IGBC comprises of 50–60% of cases of GBC and approximately 0.1–6% of all patients that undergo cholecystectomy have IGBC [25,26,27]. Conflicting reports exist regarding the survival outcomes of patients with IGBC compared to NIGBC, as some studies report that IGBC does not affect the OS, while some studies report that patients with IGBC had longer survival [25]. IGBC may represent an early stage of GBC that could progress or could represent a unique histological entity [26]. In our study, IGBC was seen in 48.9% of the patients, and cholelithiasis was more commonly found in patients with IGBC (91.3% vs. 66.7%, *p* = 0.002). T-stage and grade of differentiation were similar in patients with IGBC compared to NIGBC, although the absolute percentage of T3 tumors and poorly differentiated tumors were higher in the NIGBC group. While there were no significant differences in cumulative OS in patients with IGBC compared to NIGBC, the absolute survival was longer in patients with IGBC (2.3 years) compared to NIGBC (1.6 years) after surgical resection. In our study, the 1-year survival rate was 68.9% in IGBC compared to 68.5% in NIGBC, 3-year survival was 39.1% in IGBC compared to 36.0% in NIGBC, and 5-year survival was 35.5% in IGBC compared to 28.8% in NIGBC. The cumulative OS rates for IGBC patients in our study are comparable to a previously published study reporting 1-, 3-, and 5-year OS rates of 79.8%, 49.0%, and 40.8%, respectively [13].

There are no RCTs or large prospective cohort studies evaluating the outcomes of patients undergoing curative resection for GBC. Retrospective studies utilizing administrative databases lack individual patient data and are prone to coding errors. For these reasons, studies with access to individual patient data and accurate diagnoses through histopathology, and potential confounders for mortality such as comorbidities, remain important. The limitations of our study include the relatively small sample size and the retrospective design, information regarding the involvement of specific lymph nodes, and the limited representation of Hispanic and Asian populations in our study as compared to the general population trends in the US. Causation regarding patient characteristics and outcomes cannot be established given the retrospective nature of the study, as unmeasured confounders may be associated with the observed results and conclusions. In addition, the results and conclusions should be interpreted within the study’s context, and may not be generalizable to other patient populations with GBC. Despite the limitations, the current study enhances the literature as it provides further information on individual patient and tumor characteristics in a relatively high number of patients that underwent surgical resection with a longitudinal follow-up, and the overall survival of patients undergoing curative intent surgical intervention for GBC. Given the lack of RCTs or large-scale prospective studies, real-world data remain crucial to improve the understanding of the disease and the clinical outcomes. There is a need to improve clinical outcomes in GBC. Multicenter studies with individual patient data are needed to improve the understanding of GBC and to develop management strategies to improve outcomes for GBC. In conclusion, our results show that GBC overall has a poor prognosis, with low post-surgery OS.

## 5. Conclusions

GBC is an aggressive hepatobiliary malignancy that is often diagnosed at advanced stages. Our study shows the high rates of local and systemic involvement and high mortality. Real-world patient data remain important to identify patients at risk for worse outcomes and to stratify the risks prior to surgery.

## Figures and Tables

**Table 1:** Patient characteristics.

All Patients (*n* = 94)	*n* (%) or Median (IQR)
Female sex	58 (61.7%)
Age (years)	71 (61.8–77.0)
Race	
White	71 (75.5%)
Black	23 (24.5%)
Tumor characteristics	
Tumor size (cm)	3.3 (1.9–5.0)
Tumor histology	
Adenocarcinoma	91 (96.8%)
Squamous cell carcinoma	2 (2.1%)
Adenosquamous cell carcinoma	1 (1.1%)
Incidental GBC	46 (48.9%)
Perineural invasion	46 (48.9%)
Vascular invasion	36 (38.3%)
Positive surgical margin	47 (50%)
Tumor grade of differentiation (*n* = 92)	
Grade I (well differentiated)	7 (7.6%)
Grade II (moderately differentiated)	49 (53.3%)
Grade III (poorly differentiated)	36 (39.1%)
T-stage ^a^	
pT1a	2 (2.1%)
pT1b	11 (11.7%)
pT2	51 (54.3%)
pT3	30 (31.9%)
Treatment	
Adjuvant chemotherapy	57 (60.6%)
Adjuvant radiotherapy	16 (17.0%)
Neoadjuvant chemotherapy	3 (3.2%)
Clinical characteristics	
Cholelithiasis	74 (78.7%)
Gallbladder polyp	12 (12.8%)
Inflammatory bowel disease	3 (3.2%)
Hypertension	67 (71.3%)
Diabetes mellitus	25 (26.6%)
Tobacco use	43 (45.7%)
Alcohol use	20 (21.3%)
BMI (kg/m^2^)	28.8 (25.1–36.6)
Laboratory variables	
AST (IU/L)	35.5 (23.0–75.3)
ALT (IU/L)	33.0 (18.8–68.0)
ALP (IU/L)	102 (78.8–153)
Albumin (g/dL)	3.6 (3.1–4.1)
Total bilirubin (mg/dL)	0.7 (0.5–1.0)
Direct bilirubin	0.3 (0.2–0.65)
Creatinine (mg/dL)	0.8 (0.7–1.0)
GFR (mL/min/1.73 m^2^)	83.0 (63.0–93.0)
Hemoglobin (g/dL)	12.6 (10.9–13.5)
Platelet (×10^9^/L)	244 (193–317)
Duration from surgery to death, days (*n* = 62)	463 (166–783)

^a^ According to the AJCC 2018 TNM classification, 8th edition [14]. Abbreviations: AST, aspartate aminotransferase; ALT, alanine aminotransferase; ALP, alkaline phosphatase; BMI, body mass index; GBC, gallbladder cancer; GFR, glomerular filtration rate.

**Table 2:** Post-surgery survival characteristics.

Variables (%)	Median Survival (Years)	*p* Value (Log-Rank)
Sex		0.19
Female (61.7%)	1.7	
Male (38.3%)	2.1	
Race		0.37
White (75.5%)	1.8	
Black (24.5%)	3.0	
Tumor characteristics		
Tumor grade of differentiation		0.08
Grade I (well differentiated) (7.6%)	1.6	
Grade II (moderately differentiated) (53.3%)	2.6	
Grade III (poorly differentiated) (39.1%)	1.5	
T-stage ^a^		0.0005
T1a (2.1%) and T1b (11.7%)	4.4	
T2 (54.3%)	2.8	
T3 (31.9%)	0.9	
Vascular invasion		0.27
Present (38.3%)	1.6	
Absent (61.7%)	2.3	
Perineural invasion		0.51
Present (48.9%)	1.8	
Absent (51.1%)	2.1	
Surgical margin		0.17
Positive (50%)	1.7	
Negative (50%)	2.1	
Metastasis		0.08
Yes (54.3%)	1.6	
No (45.7%)	2.9	
Clinical characteristics		
Cholelithiasis		0.76
Yes (78.7%)	1.9	
No (21.3%)	2.1	
Gallbladder polyp		0.15
Yes (12.8%)	5.5	
No (87.2%)	1.7	
Hypertension		0.94
Yes (71.3%)	1.7	
No (28.7%)	2.3	
Diabetes mellitus		0.34
Yes (26.6%)	2.1	
No (73.4%)	1.9	
Alcohol use		0.28
Yes (21.3%)	2.8	
No (78.7%)	1.7	
BMI (kg/m^2^)		0.13
18.5–24.9 (20.2%)	1.4	
25.0–29.9 (34.0%)	1.8	
≥30 (45.7%)	2.8	
Treatment		
Adjuvant Chemotherapy		0.20
Yes (60.6%)	2.1	
No (39.4%)	1.1	
Adjuvant Radiotherapy		0.36
Yes (17%)	2.1	
No (83%)	1.9	

^a^ According to the AJCC 2018 TNM classification, 8th edition. Abbreviations: BMI, body mass index.

**Table 3:** Incidental vs. non-incidental GBC.

Variables	Incidental GBC (*n* = 46, 48.9%)	Non-Incidental GBC (*n* = 48, 51.1%)	*p* Value
Female sex	28 (60.9%)	30 (62.5%)	0.97
Age (years)	71 (64–77)	71 (61–77)	0.96
Clinical characteristics			
Cholelithiasis	42 (91.3%)	32 (66.7%)	0.002
Gallbladder polyp	4 (8.7%)	8 (16.7%)	0.25
Inflammatory bowel disease	0 (0%)	3 (6.3%)	0.08
Hypertension	30 (65.2%)	37 (77.1%)	0.21
Diabetes mellitus	12 (26.1%)	13 (27.1%)	0.91
Tobacco use	27 (58.7%)	21 (43.8%)	0.15
Alcohol use	11 (23.9%)	9 (18.8%)	0.55
Laboratory variables			
Hemoglobin (g/dL)	13 (11–14)	12 (11–14)	0.37
Platelet (×10^9^/L)	238 (202–276)	262 (185–345)	0.16
AST (IU/L)	48 (23–78)	29 (23–69)	0.50
ALT (IU/L)	34 (18–75)	32 (21–64)	0.86
ALP (IU/L)	101 (68–150)	102 (81–176)	0.72
Albumin (g/dL)	3.6 (3.2–4.1)	3.6 (3.1–4.0)	0.98
Total bilirubin (mg/dL)	0.75 (0.5–1)	0.7 (0.4–1)	0.93
Creatinine (mg/dL)	0.8 (0.7–1)	0.8 (0.6–1.1)	0.63
GFR (mL/min/1.73 m^2^)	83 (64–92)	84 (62–96)	0.97
Tumor characteristics			
Tumor size (cm)	2.7 (1.7–4.8)	4 (2–5.4)	0.47
Vascular invasion	19 (41.3%)	17 (35.4%)	1.00
Perineural invasion	26 (56.5%)	20 (41.7%)	0.69
Positive surgical margin	27 (58.7%)	20 (41.7%)	0.17
Adjuvant chemotherapy	28 (60.9%)	29 (60.4%)	0.94
Adjuvant radiotherapy	7 (15.2%)	9 (18.8%)	0.69
Death	31 (67.4%)	31 (64.6%)	0.78
Duration between surgery and death (days)	428 (164–1018)	498 (166–588)	0.14

Abbreviations: AST, aspartate aminotransferase; ALT, alanine aminotransferase; ALP, alkaline phosphatase; GFR, glomerular filtration rate.

## Data Availability

The original contributions presented in the study are included in the article/Supplementary Material, further inquiries can be directed to the corresponding author/s.

## Supplementary Materials

The following supporting information can be downloaded at: https://www.mdpi.com/article/10.3390/healthcare12202091/s1, STROBE Statement—checklist of items that should be included in reports of observational studies.
